# Three-dimensional laparoscopic surgery for colorectal cancer: A 2-year follow-up study at Hue Central Hospital

**DOI:** 10.1016/j.sopen.2023.12.007

**Published:** 2023-12-31

**Authors:** Thanh Xuan Nguyen, Nhu Hiep Pham

**Affiliations:** Department of Pediatric surgery and Abdominal emergency, Hue Central hospital, Hue city, Viet Nam

**Keywords:** 3D laparoscopic surgery, Colorectal cancer, Survival

## Abstract

**Background:**

Laparoscopic surgery has brought about a revolution in clinical practice since its inception. Using a new generation three-dimensional (3D) HD laparoscopic system can be seen as a beneficial “hybrid” created by fusing two different elements: increased vision quality and the viability and diffusion of laparoscopy. This study aims to determine the surgical outcomes and two-year survival of colorectal cancer patients after 3D laparoscopic surgery.

**Methods:**

A prospective study was conducted on 60 patients with a definitive diagnosis of primary colorectal cancer. All patients underwent 3D laparoscopic colorectal resection from January 2020 to December 2021 by a single surgical team. Data were prospectively collected from Hue Central Hospital, including operative parameters and survival time.

**Results:**

The mean age was 62.0 ± 10.6 years old. D3 lymphadenectomy accounted for most cases (96.7 %). There were no intraoperative complications or conversion to open surgery. The mean operation time was 162.3 ± 25.8 min. Postoperative complications included incisional fluid collection (5 %), wound infection (3.3 %), and drainage site bleeding (1.7 %). The average length of hospital stay was 10.4 ± 4.6 days. Overall survival rate after two years was 93 %.

**Conclusions:**

3D laparoscopic surgery for radical treatment of colorectal cancer is feasible, effective, and safe. This surgical technique offers a positive prognosis for patients.

## Introduction

Colorectal cancer is one of the four most common cancers, with about two million new cases each year and nearly one million deaths, which ranks this cancer third in terms of cancer mortality [1]. According to GLOBOCAN data, Vietnam had about 15,847 new cases of colorectal cancer and 8203 related deaths in 2020 [2]. The incidence of colorectal cancer has gradually increased worldwide in recent years [[3], [4], [5], [6], [7]]. In the current treatment of colorectal cancer, radical surgery remains the most effective treatment method [[8], [9], [10], [11]]. Since 1999, Hue Central Hospital has performed two-dimension (2D) laparoscopic colorectal surgery. However, the biggest limitation of 2D laparoscopic system is the lack of depth perception, which significantly limits surgeons' vision and tissue manipulation. Therefore, a three-dimension (3D) laparoscopic system was used to provide an enhanced 3D surgical field to facilitate orientation and depth perception.

Furthermore, 3D high-definition (HD) laparoscopy has brought transabdominal laparoscopic surgery to the next generation using high quality images [12,13]. In 2015, our hospital introduced 3D laparoscopic surgery into routine use for surgery of various types of diseases, including colorectal cancer. However, there are currently no studies in Vietnam evaluating the outcome as well as the survival of patients undergoing 3D laparoscopic surgery for colorectal cancer. Therefore, we performed this study to determine the postoperative outcomes and two-year survival of colorectal cancer patients undergoing 3D laparoscopic surgery.

## Material and methods

### Patients

Between January 2020 and December 2021, a total of 60 patients were diagnosed with colon adenocarcinoma and underwent laparoscopic surgery at Hue Central Hospital (Hue, Vietnam).

The inclusion criteria were: 1) Confirmed colorectal cancer by histopathology and complete staging using full-body computed tomography (CT) scan, endoscopic ultrasound (EUS) and/or pelvic magnetic resonance imaging (MRI). 2) All cases were discussed at tumor board conference before surgical intervention. Appropriate neoadjuvant systemic treatment for locally advanced cases was decided based on the most updated version of the National Comprehensive Cancer Network (NCCN) guidelines. 3) Preoperative anesthesia risk score American Society of Anesthesiologists (ASA) ≤ 3. The exclusion criteria included patients with intestinal obstruction, tumor perforation, or distant metastases before surgery.

All operations were performed by a single surgeon, experienced in laparoscopy and colorectal cancer surgery to avoid bias. Two other consultant surgeons served as the camera operator and assistant. Hue Central Hospital is one of three core hospitals in Vietnam that performs hundreds of colorectal cancer surgeries yearly, most of which are laparoscopic surgeries performed by the surgical team in this study. Every patient gave informed consent for surgery, and the study was conducted in accordance with the principles of the Declaration of Helsinki. The protocol was approved by the Ethical Committee of Hue Central Hospital.

### Surgical procedures

Under general anesthesia, the patients were placed in a lithotomy position. We used the Olympus HD 3D laparoscopic surgery system (Karl Storz SE & Co., Tuttlingen, Germany). The operation was performed with four trocars: one 12-mm trocar at the para-umbilicus, one 5-mm trocar at the tumor side, and one 5-mm and 12-mm trocar on the contralateral side. In right-sided colon cancer, the root of the ileocolic vein was exposed. Part of the D3 lymphadenectomy was sampled, but D3 dissection was not routinely performed. High ligation of the inferior mesenteric artery (IMA) was regularly performed for left-sided and rectal tumors. For right colectomies, we performed a hand-sewn end-to-end ileocolic anastomosis. Depending on the tumor site, a hand-sewn or circular stapled end-to-end anastomosis was used for the reconstruction in left-sided and rectal tumors.

### Evaluations

Clinical information including age, preoperative carcinoembryonic antige (CEA), ASA score, and preoperative chemotherapy was analyzed. The operative parameters were recorded, such as operative time, tumor location, number of resected lymph nodes, and intraoperative complications. The factors associated with postoperative recovery including post-operative pain, first flatus, and postoperative hospitalization were collected. The short-term postoperative complications such as anastomotic bleeding, incision infection, and bowel obstruction were investigated.

### Follow-up

The first day after surgery represented the beginning of the follow-up period. Patients were routinely followed up at outpatient clinics at 1 month, 3 months, 6 months and 12 months after discharge and every 6 months afterward. The deadline for the follow-up period was December 31, 2021.

### Statistical analysis

Statistical analysis was performed with SPSS software, version 22.0 for Windows (IBM Corporation, Armonk, NY, USA). Continuous variables are presented as the mean and standard deviations. Category variables are given as the number and percentage. Disease-free survival (DFS) and overall survival (OS) after surgery were assessed using Kaplan–Meier curvesand and the Log-rank test was used to compare the survival of the two independent groups. All *P* values were two-sided, and *P* < 0.05 was considered statistically significant.

## Results

A total of 60 patients (38 males and 22 females) were prospectively enrolled in the study, and no cases were lost to follow-up by December 31, 2021. The characteristics of the patients are summarized in Table 1. The mean age was 62.0 ± 10.6. Elevated preoperative CEA was seen in 41.7 %. Almost patients performed D3 lymphadenectomy, with the mean number of resected lymph nodes being 18.8. No cases had intraoperative complications. The operation time was 162.3 ± 25.8 mins. Post-operative pain occurred for 2.1 ± 1.4 days on average. The postoperative hospital stay was 10.4 ± 4.6 days. Postoperative complications included incisional fluid collection (5.0 %), incision infection (3.3 %), and drainage site bleeding (1.7 %).

**Table 1:** Operation-related characterestics.

Characteristics	Results
Age (year)	62.0 ± 10.6 (range: 36–90)
Elevated preoperative CEA (≥ 5 ng/ml)	25 (41.7 %)
D3 lymphadenectomy	58 (96.7 %)
Mean number of resected lymph nodes	18.8 lymph nodes
Intraoperative complications	0 %
Mean operative time	162.3 ± 25.8 mins
Post-operative pain	2.1 ± 1.4 days
Post-operative complications	
Fluid collection	3 (5.0 %)
Wound infection	2 (3.3 %)
Drainage site bleeding	1 (1.7 %)
Time to first flatus	2.1 ± 1.4 days
Length of hospital stay	10.4 ± 4.6 days

The tumors were found in all parts of the colon, from right colon to rectum; however, they were mainly located in rectum (Table 2). Post-operative complication was seen in 6 cases: two cases of perineal wound infection after abdominoperineal resection, three cases of incisional seroma at the site of specimen extraction, and one case of bleeding at the draining site. All of these complications were successfully treated with conservative treatment.

**Table 2:** Tumor locations.

Location	Results (n, %)
Right colon	9 (15.0 %)
Transverse colon	2 (3.4 %)
Left colon	5 (8.3 %)
Sigmoid colon	5 (8.3 %)
Rectum	39 (65/0 %)
Upper rectum	11 (18.4 %)
Middle rectum	14 (23.3 %)
Lower rectum	14 (23.3 %)

Table 3 shows the events that occurred within 2-year follow-up. Gastrointestinal dysfunction was found in 3 cases (5.0 %), and urinary tract infection accounted for one case (1.7 %). One case had post-operative bowel obstruction (1.7 %); five cases developed distant metastasis (8.3 %). The 12-month survival rate was 100 %.

**Table 3:** Follow-up results.

Characteristics	Results
Follow-up time (months)	18.2 ± 2.9 (range 13.2–24.1)
Gastrointestinal dysfunction	3 (5.0 %)
Urinary tract infection	1 (1.7 %)
Persisitent incisional pain	2 (3.3 %)
Post-operative bowel obstruction	1 (1.7 %)
Distant metastasis	5 (8.3 %)
12-month survival rate	100 %

**Fig. 1:**
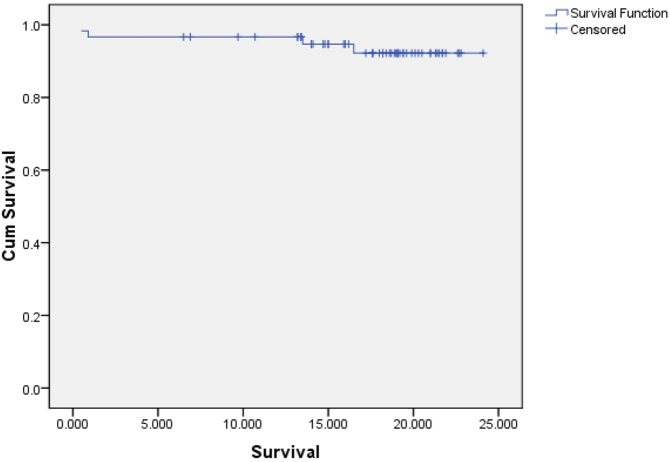
2-year overall survival rates.

The 2-year overall survival rate was 93 % (Fig. 1). Overall survival in the group of patients with stage 3 cancer was statistically significantly lower than that of stage 1 and 2 (as seen in Table 4). The difference in overall survival between groups of patients with preoperative CEA < 5 (ng/ml) and ≥ 5 (ng/ml) was not statistically significant (Table 4).

**Table 4:** 2-year overall survival rate by stage and pre-operative CEA.

	n	n after 2 years	%	*p*-Value
Stage I	15	15	100 %	0.008
Stage II	24	24	100 %	
Stage III	21	17	81 %	
CEA < 5(ng/ml)	35	34	97.1 %	0.110
CEA ≥ 5(ng/ml)	25	22	88.0 %	

**Fig. 2:**
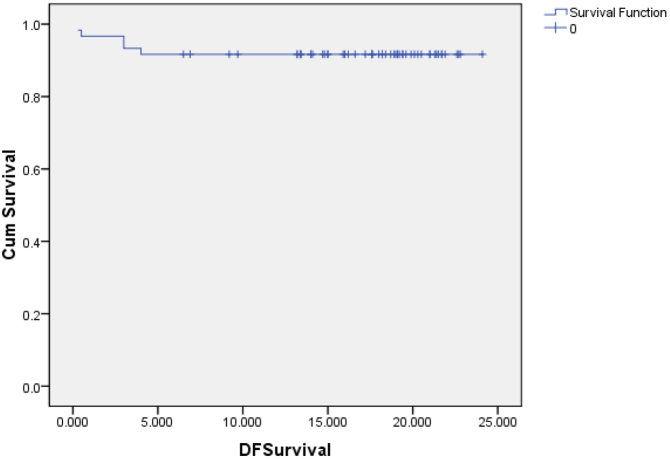
2-year disease-free survival.

The 2-year disease-free survival rate was 91.7 % (Fig. 2). The difference in disease-free survival in different stages was not statistically significant (as shown in Table 5). The difference in disease-free survival between groups of patients with preoperative CEA < 5 (ng/ml) and ≥ 5 (ng/ml) was not statistically significant (Table 5).

**Table 5:** 2-year disease-free survival by stage and pre-operative CEA.

	n	n after 2 year	%	p-Value
Stage I	15	15	100 %	0.077
Stage II	24	23	95.8 %	
Stage III	21	17	81.0 %	
CEA < 5(ng/ml)	35	33	94.3 %	0.364
CEA ≥ 5(ng/ml)	25	22	88.0 %	

## Discussion

Colorectal cancer is one of the leading causes of cancer death. In recent years, the model of colorectal cancer by age has been changing with an increasing incidence in younger patients [[14], [15], [16], [17]]. From January 2020 to December 2021, 60 patients were radically treated for colorectal cancer by 3D laparoscopic surgery with a mean age of 62.0 ± 10.6 (36–90) years. In our study, rectal tumors accounted for the majority of cases (65 %), followed by right colon (15 %), left colon (8.3 %), and sigmoid colon (8.3 %). The extent of lymphadenectomy was D3 (96.7 %) and D2 (3.3 %). The Union for International Cancer Control clearly states that accurate pathological staging requires >12 lymph nodes to be examined in resected specimens [18]. In this study, the mean number of lymph nodes removed was 18.8 nodes, of which the number of patients with >20 lymph nodes harvested was 21 (35 %) patients.

In our study, there were no cases that needed to be converted to open surgery and no major complications during surgery. According to the study by Hewett, the rate of anastomotic leakage was 1.4 % [19]. In one study, the reported intraoperative complication rate was 4 %, and a postoperative complication rate was as high as 19 % [20]. The advent of the 3D laparoscopic system resulted in significant improvement in depth perception, spatial location, and resolution compared to the conventional 2D laparoscopic imaging system. As a result, nearby structures and blood vessels can be easily visualized and, at the same time, the possibility of injury, bleeding, and surgical complications is reduced [21]. This is especially true in rectal surgery where the 3D system provides clearer anatomic images of the pelvis to facilitate nerve preservation and thereby may reduce the risk of seminal vesicles and posterior vaginal wall injury [22].

Comparative studies of 3D and 2D laparoscopic surgery in gastrointestinal tumors has been performed, demonstrating that 3D laparoscopic surgery can improve the spatial position and depth of manipulation, reduce the difficulty of fine manipulation and shorten the operative time [21,22]. A previous study reported significantly shorter operative time and less bleeding observed in 3D laparoscopic surgery compared with its 2D counterpart [23]. In our study, the mean operative time was 162.3 ± 25.8 min, in which the fastest operative time was 120 min and the longest operative time was 270 min. This duration is shorter than that as reported in a systematic review and meta-analysis by Pantalos [24]. Many studies comparing operative time between 3D and 2D also recorded a significantly shorter operative time in the 3D group compared to 2D group [22,25]. Reducing operative time helps avoid the risks of prolonged anesthesia and hastens the recovery period.

Regarding postoperative results, the mean postoperative pain time was 2.1 ± 1.4 days, the mean recovery time was 2.1 ± 1.4 days and the mean hospital stay was 10.4 ± 4.6 days (6–26 days). Considering post-operative complications, only six patients (10 %) had postoperative complications, including two cases of perineal wound infection after abdominoperineal resection, three cases of incisional seroma at the site of specimen extraction, and one case of bleeding at the draining site were all successfully managed conservatively. There was no anastomotic leakage requiring reoperation. Su argued that compared with 2D endoscopy, tissue retraction, dissection, hemostasis, and vessel ligation were easier because 3D laparoscopy provides the surgeon with better depth of field and hand-eye coordination [26]. thereby helping to limit postoperative complications. Zeng also confirmed that 3D laparoscopic surgery is more effective in treating rectal cancer than 2D laparoscopic surgery. The training curve in laparoscopic colectomy is achieved in 20–50 cases, depending on experience and skills in laparoscopic manipulation, especially in colorectal surgery [22].

The length of stay was around 10 days when time to bowel function was 2 days. This is usually seen in Vietnam. Medically, the patient can be discharged sooner. But in our social security systems, patients must wait for the definite pathology followed by an multidisciplinary teams before the discharge. If not, patients can not have the cover of social security for the next treatment (oncology treatment).

Regarding the survival rate, Hong et al. in their publication in 2021, recorded the overall survival (OS) and disease-free survival (DFS) of colorectal cancer patients after 5 years of 79.5 % and 69.5 %, respectively [27]. Another single-center study published in 2007 reported that the overall survival and disease-free survival of colorectal cancer patients were 71 % and 61 %, respectively [28]. The report of Joachim et al. in 2019 showed less satisfactory results with OS after 5 years of only 43.8 % [29]. These results are somewhat similar to our study. Although our postoperative follow-up time was shorter, the OS and DFS after 24 months were still >80 %. Our patients had a 12-month survival rate of 100 %, better than that of Joachim et al. of only 74.6 % [29]. Our result is not favorable because our study group had a higher proportion of patients who presented at later stages with large tumors. Similar results with less than optimal survival prognosis in patients with late stages were reported in the literature [27,28]. Late complications only presented in 7/60 patients, of which there was only one case of intestinal obstruction, while the rest were only mild functional disorders.

We had 8.3 % cases presenting with distant metastases after 24 months of follow-up, while there was no local recurrence. Lee et al. recorded the overall recurrence rate (including metastasis) up to 19 %, of which 2.4 % was a local recurrence. Although our follow-up period was not long enough, the results are encouraging. Local recurrence is often related to surgical techniques, including failure to achieve a wide resection margin or sufficient lymph node dissection. Perhaps the role of 3D laparoscopy in improving the radicality of surgery in our study helped to reduce the local recurrence rate. However, to confirm this, it will be necessary to extend our follow-up period.

Studies in the literature have shown a correlation between CEA levels and patient survival, with the group of patients having higher preoperative CEA has poorer prognosis [[27], [28], [29]]. Our analysis also showed similar results, with the group of patients with serum CEA levels lower than 5 ng/ml having a statistically significant higher survival rate.

Our study study has some limitations such as small size sample, absence of a control group to make a comparison between 3D and 2D laparoscopic surgery, and short-term follow-up. However, this is the first prospective study evaluating the benefit of 3D laparoscopic colectomy for colon cancer in Vietnam.

## Conclusions

3D laparoscopy for the radical treatment of colorectal cancer is a feasible, effective, safe method and offers a positive prognosis for patients. More multicenter randomized prospective studies are needed to compare the effects of 3D vs. 2D laparoscopic radical resection of rectal cancer.

## Ethics statement

The study was approved by the Ethics Committee of the Hue Central Hospital according to the principles outlined in the Declaration of Helsinki. Written informed consent was obtained from all study subjects or their legal guardians. Participation was voluntary for all participants and all the participants had the right to withdraw at any time.

## Funding

This is the result of the Provincial Science and Technology project invested by the state budget of Thua Thien Hue province (TTH.2018-KC.11).

## CRediT authorship contribution statement

**Thanh Xuan Nguyen:** Conceptualization, Data curation, Formal analysis, Funding acquisition, Investigation, Methodology, Project administration, Resources, Software, Supervision, Validation, Visualization, Writing – original draft, Writing – review & editing. **Nhu Hiep Pham:** Conceptualization, Data curation, Formal analysis, Funding acquisition, Investigation, Methodology, Project administration, Resources, Software, Supervision, Validation, Visualization, Writing – original draft, Writing – review & editing.

## Declaration of competing interest

The authors declare that they have no known competing financial interests or personal relationships that could have appeared to influence the work reported in this paper.
